# A new approach for fabrications of SiC based photodetectors

**DOI:** 10.1038/srep23457

**Published:** 2016-03-18

**Authors:** Ali Aldalbahi, Eric Li, Manuel Rivera, Rafael Velazquez, Tariq Altalhi, Xiaoyan Peng, Peter X. Feng

**Affiliations:** 1 Department of Chemistry, College of Science, King Saud University, Riyadh 11451, Saudi Arabia; 2 King Abdullah Institute for Nanotechnology, King Saud University, Riyadh 11451, Saudi Arabia; 3 Department of Physics, College of Natural Sciences, University of Puerto Rico, San Juan, 00936-8377, PR/USA; 4 Department of Chemistry, Faculty of Science , Taif University, Saudi Arabia; 5 College of Electronic and Information Engineering, Southwest University, Chongqing, 400714, China

## Abstract

We report on a new approach to quickly synthesize high-quality single crystalline wide band gap silicon carbide (SiC) films for development of high-performance deep ultraviolet (UV) photodetectors. The fabricated SiC based UV photodetectors exhibited high response while maintaining cost-effectiveness and size miniaturization. Focus of the experiments was on studies of electrical and electronic properties, as well as responsivity, response and recovery times, and repeatability of the deep UV photodetectors. Raman scattering spectroscopy and scanning electron microscope (SEM) were used to characterize the SiC materials. Analyses of the SEM data indicated that highly flat SiC thin films have been obtained. Based on the synthesized SiC, deep UV detectors are designed, fabricated, and tested with various UV wavelength lights at different radiation intensities. Temperature effect and bias effect on the photocurrent strength and signal-to-noise ratio, humidity effect on the response time and recovery time of the fabricated detectors have been carefully characterized and discussed. The detectors appear to have a very stable baseline and repeatability. The obtained responsivity is more than 40% higher compared to commercial detectors. The good performance of the photodetectors at operating temperature up to 300 °C remains nearly unchanged.

## Introduction

Semiconductor based UV photodetectors can normally be classified into 4 types: photoconductors, metal-semiconductor-metal (MSM) diodes, Schottky barrier and p-i-n photodiodes^1^. The MSM diode is made of two Schottky barrier diodes back-to-back. The entire semiconductor layer between two metal electrodes becomes fully depleted under sufficient bias. The geometric parameters of a conventional MSM photodetector pixel element determines its performance. The device consists of alternating metal contacts deposited on the semiconductor active layer. MSM structures represent a very simple photodetector design and its performance is being continuously improved by the incorporation of novel synthesized semiconductor materials. In recent years, various wide band gap semiconductors such as diamond^2^,^3^, nitride (AlN, GaN, BN)^4^,^5^,^6^,^7^,^8^,^9^, and oxide (TiO_2_, ZnO)^10^,^11^ semiconducting materials have been investigated for their potential application as sensing materials in deep UV photodetectors.

Silicon carbide (SiC) is also considered one of the most important wide band gap materials in the development of UV photodetectors. SiC based photodetectors can achieve large gains, high signal-to-noise ratios and solar-blind operation. This makes them ideal photodetectors in certain applications for monitoring the UV spectrum without the need for solar rejection filters. Furthermore, semiconductor SiC offers outstanding long term stability even when operated under high-intensity UV radiation (up to 1000 W/m^2^) and at high operating temperature^12^,^13^. For example, using rapid thermal chemical vapor deposition (RTCVD) techniques, Chang, *et al*. developed a hetero-epitaxial SiC/Si MSM photodetector for high-temperature deep-UV detecting applications^14^. The optoelectron performances of the SiC-MSM photodetectors were examined by the measurement of photo and dark currents and their ratio under various operating temperatures. The current ratio for 254 nm UV light of the detector is about 6.5 at room temperature and 2.3 at 200 °C. Lien, *et al*. reported on high-temperature operation of MSM photodetectors using low-temperature, ion beam-assisted deposition of nanocrystalline SiC thin films and hydrothermal synthesis of ZnO nanorod arrays (NRAs)^15^. Due to the incorporation of ZnO NRAs, the photo-to-dark current ratio of SiC MSM photodetectors increased from 4.9 to 13.3 at 25 °C and from 4.9 to 7.6 at 200 °C. The enhancement in the sensitivity suggested that the ZnO NRAs could serve as an effective antireflective layer allowing more light into the SiC MSM photodetectors. Chen, *et al*. theoretically investigated the temperature dependence of current-voltage and spectral response characteristics of a 4H-SiC MSM UV photodetector in the temperature range from room temperature to 800 K with two-dimensional (2D) numerical simulator^16^. Computation simulation indicated that the dark current and photocurrent increased with an increase of temperature. For the range of 500–800 K, the dark current increases by nearly a factor 3.5 every 150 K larger than that of photocurrent, leading to a negative effect on photodetector current ratio.

Besides MSM architectures, Prasai, *et al*. developed 4H-SiC p–n diodes based UV light detection^17^. The behavior of the photocurrent response under UV light irradiation using a low-pressure mercury UV-C lamp (4 mW/cm^2^) and a medium-pressure mercury discharge lamp (17 mW/cm^2^) was studied. The devices under test showed an initial burn-in effect, i.e., the photocurrent response dropped by less than 5% within the first 40 h of artificial UV aging. Such burn-in effect under UV stress was also observed for previously available 6H silicon carbide (6H–SiC) p–n photodetectors^17^. After burn-in, no measurable degradation was detected, which makes the devices excellent candidates for high irradiance UV detector applications. Anderson, *et al*. used graphene/SiC hetero junction to develop a new UV detector^18^. They exploited the 2D nature of graphene to minimize absorption losses for high-efficiency sensing while simultaneously taking advantage of the epitaxial p–n junction to achieve low reverse leakage. A quantum efficiency above 80% at 4 eV was obtained.

Recently, new deep UV avalanche photodiodes (DUVAP) have been developed and have resulted in significant progress in UV photodetector research^19^,^20^. The higher quantum efficiency of Geiger-mode avalanche photodiode (GM-APD) detectors and the ability to fabricate arrays of individually-addressable detectors, opens up a wide range of applications requiring deep UV detection. A. Akturk *et al*. used simulators for the SiC APDs in conjunction with special fabrication techniques to develop a recipe for the fabrication of APD chips^19^. One of the problems associated with deep-UV light detection is that the absorption coefficient of this wavelength in SiC is large. Therefore the electron-hole creation occurs near the surface where there is a minimal electric field to drift newly generated carriers. To increase efficiency and responsivity, surface recombination must be reduced. This is achieved by either passivating recombination centers at the surface or increasing the field at the surface, which results in electron-hole separation before any possible recombination. Shaw, *et al*. discussed the system design trades and precise microelectronic technology to develop deep UV sensing GM-APD arrays, and presented preliminary performance data for their silicon carbide GM-APD arrays^20^. The obtained responsivity is up to 0.13 amp photocurrent per Watt of light power (A/W), and operating temperature can be up to 150 °C. However, fabrication of high performance SiC UV photodetector is challenging due to some material defects, and relatively not-well modeled device operation, etc. Sang^1^, Goldsman^21^, Edmond^22^, and Omnes^23^ systematically and comprehensively reviewed various types of semiconductors based deep UV photodetectors.

The objective of this work is to extend the state-of-the-art in UV sensors by: a) using a new approach to synthesize high quality of SiC film, b) developing unique fabrication techniques improve surface quality of the SiC deep UV detectors, and c) improving their responsivity and performance at high temperature. Ultra-thin nano Schottky contact is employed in the present work in order to have high efficiency. A simple and quick approach is used to fast fabrication of prototype. A goal is to develop a small, low-cost, highly reliable UV detector with high responsivity. The basic properties of the prototypes such as responsivity, response time, recovery time, repeatability, and stability have carefully been characterized. The effect of humidity on the detector has also been discussed and estimated. The obtained detectors appear to have a very stable baseline, and repeatability. A responsivity up to 0.18 A/W was obtained. Even when the operating temperature is as high as 300 °C, the fabricated detectors still displayed good performance in responsivity, stability, and repeatability.

### Synthesis of SiC thin films

The SiC used as sensing material was synthesized by using laser plasma deposition system. The details of the process is described in our previous publication^6^. Briefly, the laser beam, focused with a 30 cm focal length of ZnSe lens, was incident at 45 degree angle relative to a rotated (speed of circa 200 rpm) pyrolytic SiC target under high vacuum (2.66 × 10^−3^ Pa) chamber. The diameter of the focus spot of the laser beam on the target was about 2 mm that could be varied by shifting focal lens. The power density of the laser on the target was 2 × 10^8^ W/cm^2^ per pulse. Metal gold and Silicon (Si) wafers (1 × 1 cm^2^) were used as substrates and placed 5 cm away from the target. Substrate temperature was kept at ∼700 °C for 60 minutes. Prior to the experiments, the substrates were ultrasonically washed in the methanol solution for 5 minutes, and dried with helium gas.

**Figure 1:**
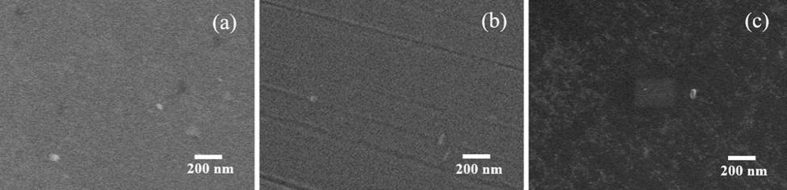
SEM images of SiC films prepared on (**a**) Si and (**b**) Au substrates, respectively. (**c**) SEM image of SiC/Si after surface treatment with Pt nanoparticles.

Figure 1 shows SEM images of the obtained SiC samples prepared on (a) Si and (b) Au substrates, respectively. The thickness of the film is around 2 μm. The obtained samples appear highly flat and smooth surface together with a few nano particles with average grain size from 300 to 500 nm. Several tracks from the surface of SiC/Au can be observed and are due to the non- uniform surface of the metal substrate.

**Figure 2:**
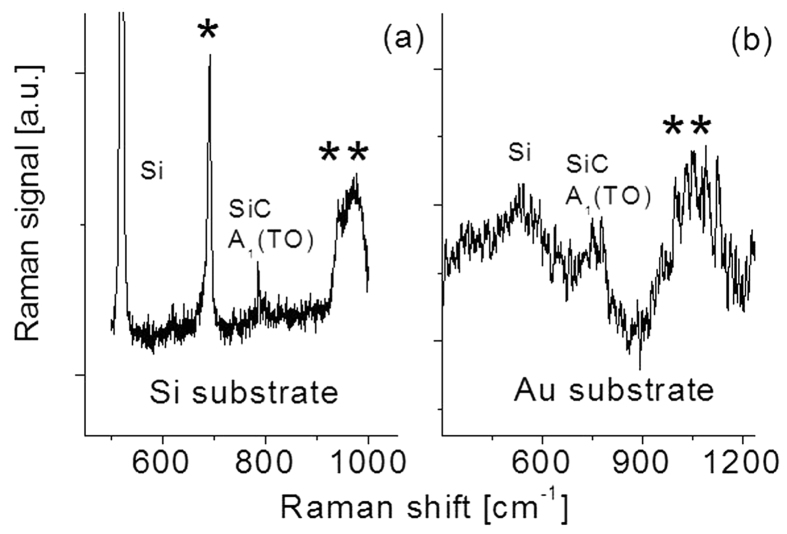
Raman scattering spectra of SiC films prepared on (**a**) Si and (**b**) Au substrates.

Both SiC/Si and SiC/Au samples are analysed by Raman scattering spectroscopy using triple-gratings monochromator with an excitation wavelength of 514 nm from an Ar^+^ ion laser. Three slits with widths of 150 μm, 27648 μm, and 150 μm were used in laser beam collimation. The laser power is maintained around 2 ~ 4 mW. An Olympus microscope with 80× microscope objective focuses the laser beam to give a spot size of 3 ~ 4 μm in diameter on the sample surface, yielding power density around 2 × 10^4^ W/cm^2^. Total accumulation time is 30 seconds for the measurement. It has been found that the morphology of the surface of the sample remains unchanged before and after Raman spectral measurements, indicating there is no obvious annealing effect on the sample at the condition of laser power density around 2 × 10^4^ W/cm^2^ with accumulation time of 30 seconds. Figure 2 shows Raman spectra of both SiC samples. In Fig. 2a, the strongest peak at about 521 cm^−1^ is due to the Si substrate. The peak at 790 cm^−1^ corresponds to SiCA_1_ (TO) phonon. A broad band marked with two asterisks in SiC spectrum is also observed, which is attributed to 2TO of SiC together with Si substrate contribution^24^.

A peak at about 690 cm^−1^ marked with an asterisk is also observed. We expect that the origin of this Raman band is from tungsten oxide (WO) impurity. Since heating samples during the deposition was based on a hot tungsten wire. Oxygen gas was from residue gas inside the vacuum chamber. Once chemical reaction between the tungsten and the oxygen occurs, the formation of WO is easy to vaporize at high temperature and some particles would reach to the surface of the sample. Energy Dispersive X-Ray Spectroscopy (EDX) has been used to confirm this phenomenon.

For SiC/Au case, SiC Raman peaks can also be easily identified. Referring Raman spectrum of the sample 1 and literature, the SiC peak should be around 790 cm^−1^. But in the present case, it appears at 785 cm^−1^, red shifted by 5 cm^−1^. The large red shift is due to metal substrate thermal expansion induced by hot laser plasma during the deposition, resulting in huge stress/strain at the interface. A broad band related to Si was also observed, indicating the synthesized SiC sample consists of both Si and SiC components.

It is easy to find that intensities of the Raman spectral lines from SiC/Si and SiC/Au are different. The relative quenching in the Raman bands of SiC/Au with respect to SiC/Si is most likely due to the background reflection from the Au substrate. Several factors such as crystal structure, weak laser power, super thin film or surface roughness, etc. would affect Raman signal. The film (2 μm) is considerably thick for a Raman study. After comparison of previous Raman measurements of SiC with various thickness (0.2–30 μm) on Si substrate, the Raman signal ratio between these from the SiC sample and the Si substrate in the present case is in good agreement with the results published by other groups^25^,^26^. Analyses of our EDX data indicated that the content of carbon atoms are slightly larger than Si content, and C/Si atomic ratio is around 1.07, indicating that the synthesized films of SiC might be associated with few defects or an amorphous Si_x_C_1−x_ alloy. Nevertheless, a narrow SiC Raman spectral line strongly suggests that high-quality SiC films have been obtained.

From a comparison of Raman spectral lines in Fig. 2, we may conclude that the crystalline quality of SiC/Si is relatively better than that of SiC/Au. Therefore, in the following report, the main work on development of deep UV detectors was based on SiC/Si samples.

### Device processing and electrical properties

**Figure 3:**
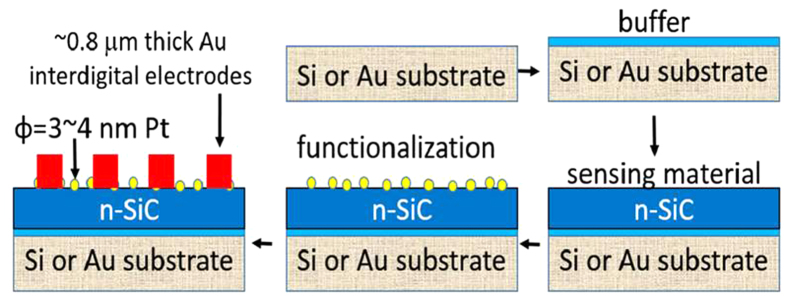
Process flow for fabrication of SiC UV photodetector. Cross-section of SiC photodetector with “ultra-thin” Schottky contact. The SiC thickness is around 2 μm and the buffer layer thickness is around 5 nm (not to scale).

To explore the possibility of using the SiC material for device applications, the SiC samples were functionalized using platinum (Pt) nanoparticles prior to measurements. The surface treatments with Pt nanoparticles for 30 seconds was performed by using 200 W Radio Frequency (RF) Magnetron Sputtering deposition technique. The process flow for the sensing material fabrication is shown in Fig. 3. The thickness of the SiC film is 2 μm and buffer layer thickness is around 5 nm. The purpose we used “buffer” layer is to reduce the stress between the substrate and the SiC. However after the comparison of two, no obvious difference in performance was observed. The architecture of the photodetector we used is similar to our previous boron nitride work^6^ but we here used a completely different SiC sensing material. It should be mentioned that an “ultra-thin” (3 ~ 4 nm Pt) Schottky contact has been employed in the present work in order to have a high efficiency^27^,^28^. Once the surface treatment was completed, prototypic deep UV photodetector was fabricated.

A mask is placed over the top surface of the SiC sample, and the Au metal (interdigital electrodes with thickness of 0.5 ~ 1 μm) is directly coated (for 2 minutes) onto the platform. The prototype is then annealed at 200 °C for 60 minutes. This is a very simple and quick approach for fast fabrications. The gap between a pair of electrical electrodes is 400 μm. Smaller gaps can be obtained if lithography is used but the process is much more lengthy and complicated; typically involving design, resist coating, exposure, etching, development, and so on. The electrode architecture employed in the current work proves to be much simpler, faster to produce, and economical, than other conventional structures while retaining performance quality.

The photon-induced electrical current *I*_ph_ observed when the sensing material is exposed to UV radiation is generated by incoming photons that excite electrons from the valence to conduction band and some carriers are collected by a pair of electrodes. The electrical current is given by *I*_ph_ = GηAgΦ^29^ where *G* is the photoconductive gain, η is the quantum efficiency, *q* is the electronic charge, *A* is the device area exposed to incident radiation, and Φ is the incident flux. The photoconductive gain and the quantum efficiency are the most important parameters influenced by the material characteristics.

**Figure 4:**
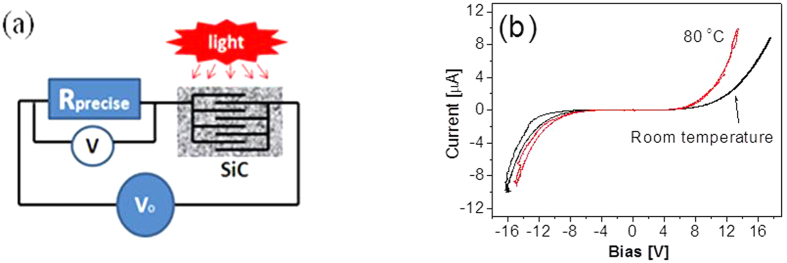
(**a**) Schematic of experimental set up for characterization of detector’s electric properties and light responsivity; (**b**) Typical dark current of SiC based UV photodetector as a function of the applied bias voltage.

Electrical properties of SiC sensing materials were characterized by using an Agilent voltage source power supply, and two HEWLETT 34401 electrical multimeters controlled by Labview program. Figure 4a show schematic diagram of experimental set up for characterization of detector’s electrical properties. The error of the measurements was around 5%. The prototype is serially connected to a precise resistor R_p_, a switch, and power supply V_o_ with step voltage. An electrical meter (V) was used to measure voltage variations during the characterization of the fabricated detectors. A thermocouple and tungsten filament was used to control operating temperature. At a given temperature (T_o_), the electrical current and voltage curves at different temperatures were obtained. Figure 4b shows typical dark current of the detector as a function of the applied bias voltage where the bias varies from zero voltage to 16 V, and then to −16 V, and then return to zero voltage for each measurement. The dark current is less than 0.12 μA at the applied voltage of 5 V and 0.8 μA at 10 V at room temperature. An increase of temperature up to 80 °C results in an increase of the dark current from 0.2 μA at 5 V to 2.5 μA at 10 V. The slightly high dark current for the fabricated detector is possibly due to doping of SiC that is used in order to achieve high responsivity. These characteristics are directly attributed to material properties.

### Characterizations of the fabricated photodetector’s responsivity

**Figure 5:**
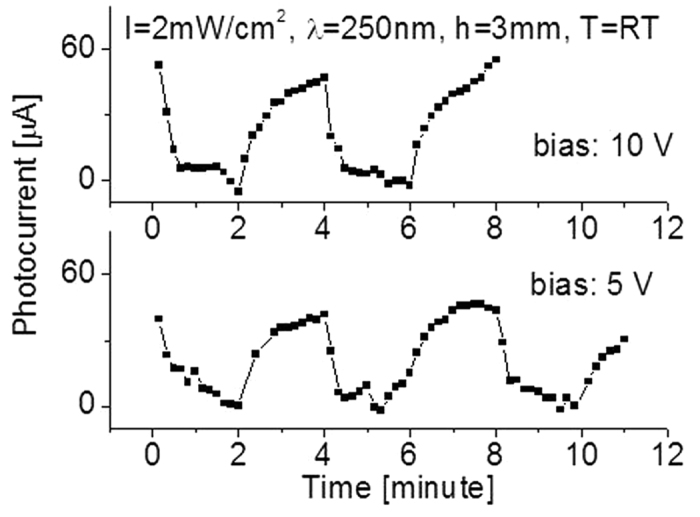
Bias effect on the response of the detector to λ = 250 nm light with intensity of I = 2 mW/cm^2^ at room temperature (T = RT).

Pen-Ray Deep UV lamps with different wavelengths produced by UVP, LLC were used as light sources for the characterization of the SiC-based deep UV photodetectors. Light intensity on the surface of the detector was controlled by the change of the distance (h) between the detector and the UV lamp. Prior to any UV radiation sensitivity measurements of the photodetector, the effect of bias voltage on the detector was characterized. Figure 5 shows the typical photocurrent response of the detector to a UV light source at different bias.

Slight differences can be easily identified. Normally, high bias yields high response or photocurrent. At room temperature the yielded photocurrent (A) was A = 55 micro amps (μA) at bias of 10 V, and A = 45 μA at 5 V when exposed to 250 nm light lamp with intensity of 2 mW/cm^2^. Therefore, in the following characterizations, a bias of 10 V was used for all cases. As is seen in Fig. 5, the output baseline of the prototypic detector appears to be very stable and well defined.

**Figure 6:**
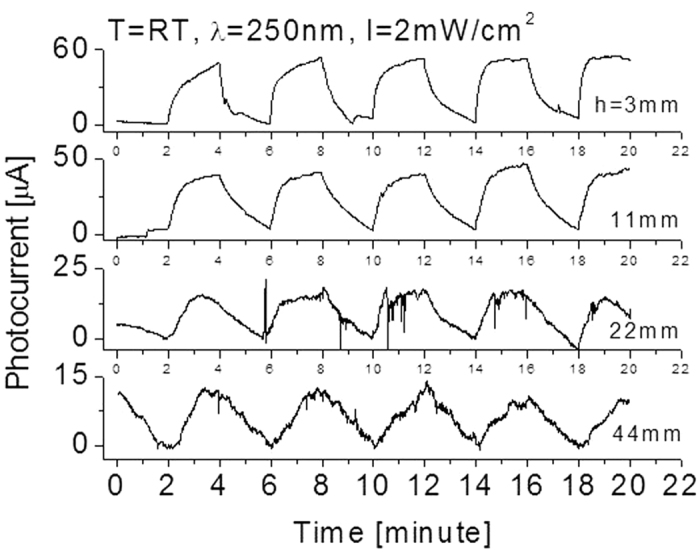
Responses at room temperatures when the prototype was cycled with a period of 4 minutes between the “switch-on” and “switch-off” of the 250 nm UV light sources with different intensities. T: temperature; λ: wavelength; I: light intensity; h: the distance between the detector and the light lamp.

The characterizations of the detector properties include the responsivity (RE), the response time (t_resp_), the recovery time (t_rec_) and the photocurrent (A). The repeatability and stability are the other two important parameters for a new detector. Figure 6 shows typical responses of the detector when different radiation intensities were applied and operated at room temperatures. The photodetector was cycled with a period of 4 minutes between the “switch-on” and “switch-off” of a 250 nm UV light source. When the detector was exposed to light, the yielded photocurrent increased and then reached a relatively stable value. When the light was switched off, the photocurrent decreased and then gradually reached zero value. It is clear from Fig. 6 that the detector has good features in repeatability and stability. Clearly, the yielded photocurrent can be attributed to the absorption of UV photon. 2 mW/cm^2^ of 250 nm light intensity yielded around A = 55 μA photocurrent from the deep UV photodetector located at the distance h = 3 mm away from the light source, and a photocurrent of 47 μA, 21 μA, and 14 μA at h = 11 mm, 22 mm, and 44 mm, respectively. The photocurrent strength decreases with an increase of the distance (h) between the detector and the light source. This is because of the decreasing light intensity on the surface of the detector. Experimental data in Fig. 6 also indicate that the signal-to-noise ratio decreases with a decrease of incident light radiation intensity on the surface of the detector.

The responsivity (RE) is defined as RE = A_photocurrent_/W_light_ where A_photocurrent_ is the maximal photocurrent from the detector, and W_light_ is the total input of light power on the surface of the detector. Since the exposure surface area of the detector is 15 mm^2^, 250 nm light power on the surface the detector is around 300 μW. Equivalently, there are total 3.9 × 10^14^ photons per second on the surface of the detector. From obtained experimental data, we observed that the maximal photocurrent in the detector’s output is 55 μA or 3.3 × 10^14^ electrons per second. This indicated that 100 photons would yield around 86 electrons. High photoelectron yield in the deep UV photodetector is probably related to its high quantum efficiency^27^,^28^,^30^. In fact, it is found that the obtained responsivity of RE_exp_ = 55 μA/300 μW = 0.18 A/W at the bias of 10 V is much larger than that of the existing detectors developed by other groups^27^,^30^.

It was also found from Fig. 6 that the response and recovery times on each cycle slightly varied. The definition of response time was based on the time duration for reaching 90% of the full response of the detector. Several work including ours were performed on studies of response and recovery times of photodetectors but the focus of the most work is on ZnO or boron nitride based photo detectors^6^,^31^,^32^. For the present SiC case, the response times at the first several cycles are long and become shorter as more cycles are completed. For example, the response time was t_resp_ = 90 seconds at the first cycle and 60 seconds, 42 seconds, 27 seconds and 18 seconds for the 2^nd^, 3^rd^, 4^th^ and 5^th^ cycle, respectively. Very slight improvement for the recovery time was also found.

**Figure 7:**
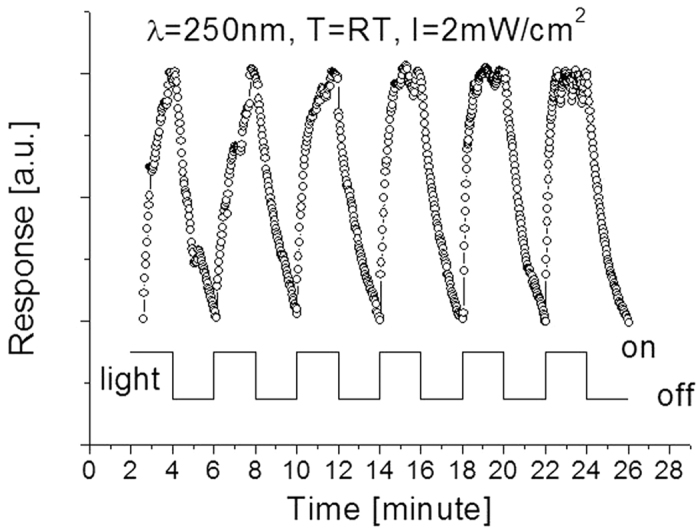
Responses at room temperatures when the prototype was cycled with a period of 4 minutes between the “switch-on” and “switch-off” of the 250 nm UV light sources at h = 3 mm.

The phenomena above could be attributed to humidity effect. In order to understand this effect, the SiC photodetector is re-characterized after two weeks of exposure in open air where humidity level is relatively high because of tropical weather. The obtained response evolution is shown in Fig. 7, from which one can easily find that the time response varies following the cycling but the photodetector appears to have good features in repeatability and stability.

At the very beginning of few cycling tests, the response time was poor, around t_resp_ = 120 seconds. It became better/shorter after several cycles of UV light radiation as 108, 39, 30, and 14 seconds at the 2^nd^, 4^th^, 5^th^ and 6^th^ cycle, respectively.

**Figure 8:**
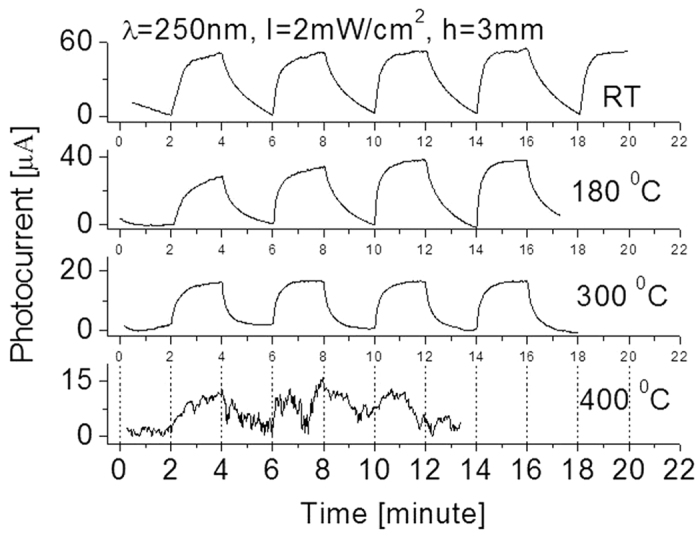
Temperature effect on the response of the detector to λ = 250 nm light source with intensity of 2 mW/cm^2^ at h = 3 mm.

A tentative interpretation is that environmental impure or humidity such as water molecules are possible factor influencing the performance of the detector. Water adsorbed on the SiC surface would not donate electrons to sensing layers but it would partially absorbed light. This would lower the responsivity of SiC detector. The reaction between the sensing material surface molecule and the water molecules would lead to the gradual formation of stable chemical bond on the surface, causing a progressive deterioration of the responsivity of the detector. However, once the detector was exposed to deep UV light source, it caused vaporization of partial impurities from the surface. Humidity effect on the properties of the sensitivity and response time of the detector relies on the competitive surface effects resulted from adsorption/desorption and the related ionization/dissociation of water molecules on the photo-generated charge carriers. Recently, Lai, *et al*. reported their study of humidity effect on ZnO based the ultraviolet sensors^33^. Subsequent heating could avoid a humidity effect on the properties of deep UV photodetectors. Therefore, additional experiments at different operating temperatures have also been carried out and the results are shown in Fig. 8. It was interesting that a temperature effect on the performance of the fabricated photodetector was observed. Such effect was preliminarily studied by other group before^15^. As seen from Fig. 8 following an increase of operating temperature from 25 to 180 and then to 300 °C, the yielded photocurrent decreases from 55 to 38, and then to 16 μA. Both response time and recovery time were improved at high operating temperature as compared to that at room temperature. Even if operated at 300 °C, the fabricated detector still runs well with good features in stability and repeatability. These characteristics are much better than that of commercial SiC detectors that normally operate at temperatures less than 150 °C. However, once the operating temperature is at 400 °C, the thermal noise nearly dominates the fabricated photodetector output. At this moment, it is difficult to identify the real signal from the detector output as weak light signal has completely been merged into strong thermal noise.

**Figure 9:**
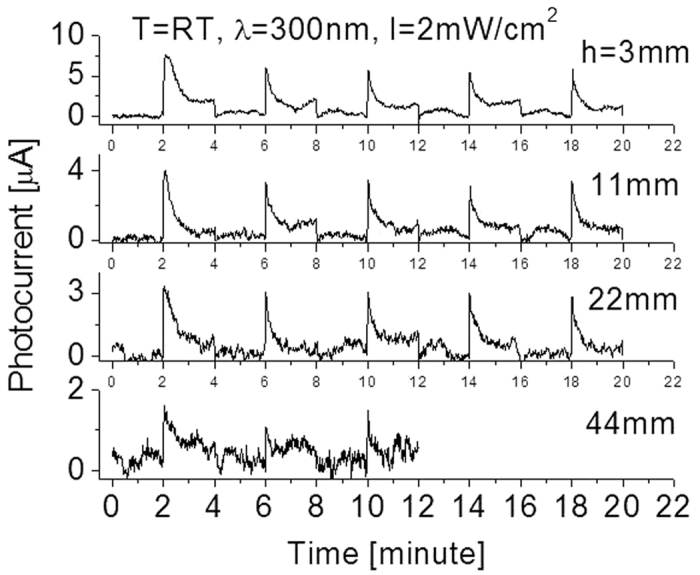
Responses at room temperatures when the prototype was cycled with a period of 4 minutes between the “switch-on” and “switch-off” of the 300 nm UV light sources with different intensities (due to variation of the parameter “h”).

As a comparison, characterizations of the prototype exposed to other UV wavelengths of lights were also conducted. Fig. 9 shows responses at room temperatures when the prototype was cycled with a period of 4 minutes between the “switch-on” and “switch-off” of a 300 nm UV light source with different intensities. High stability or repeatability features are clearly visible. However, the response strength or the photocurrent of the detector exposed to 300 nm light was almost 7–8 times less than that to 250 nm light at the same radiation intensity, indicating the fabricated SiC based detector is highly sensitive to a short wavelength of UV light. The feature of the responsivity relies on semiconductor electronic properties such as bandgap width and band structures. The obtained results are in good agreement with previously reported experimental data^22^.

Furthermore, the evolution of the response time obtained from the cyclical test by using 300 nm was also obviously different from that of the detector exposed to 250 nm light. The photocurrent quickly jumped to its peak, and then gradually decreased and reached its stable value when 300 nm UV light with intensity of 2 mW/cm^2^ (at h = 3 mm) incident radiation was used. The obtained photocurrent is around A_peak_ = 7.5 μA at peak and A_stable_ = 2 μA at stable state when the SiC detector is located at distance h = 3 mm away from the lamp, and A_peak_ = 7.3 μA, A_stable_ = 1.8 μA at h = 11 mm, and A_peak_ = 5.5 μA, A_stable_ = 1.0 μA at h = 22 mm, and A_peak_ = 2.0 μA, A_stable_ = 0.6 μA at h = 44 mm. Since the light power on the surface of the detector (located h = 3 mm away from the lamp) was 300 μW, we obtained the responsivity of RE_peak_ = 7.3 μA/300 μW = 0.024 A/W at peak and RE_stable_ = 0.0067 A/W at stable state. Experimental data also indicated that if the light source (2 mW/cm^2^) was placed more than 44 mm away from the detector or if the incident light radiation strength was too low, very weak photocurrent would be created that was generally merged into noise signal as shown in Fig. 9. When the light was switched off, the photocurrent from the detector steeply dropped to zero. The graph of the output signal has very steep slopes at the moment of turning on or off the light, indicating a fast response time and recovery time.

**Figure 10:**
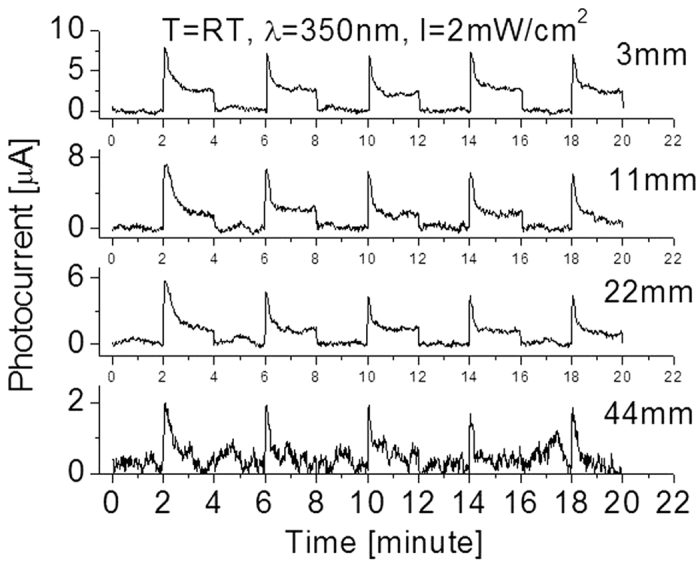
Responses at room temperatures when the prototype was cycled with a period of 4 minutes between the “switch-on” and “switch-off” of the 300 nm UV light sources with different intensities.

Similar phenomenon was also found when the detector was exposed to 350 nm light source as shown in Fig. 10. The obtained experimental data including photocurrent, response time, and recovery time were all similar to that of the detector exposed to 300 nm light. Prompt increase of the photocurrent at very beginning and quick drop at the end of each cycle are clearly visible. Once the light lamp was moved to 44 mm away from the detector or the incident light radiation of power dropped down to few micro watt per centimeter square, nearly no response could be detected except for the signal at the instant of turning on and off the light. High stable baseline and excellent repeatability can be easily identified.

**Figure 11:**
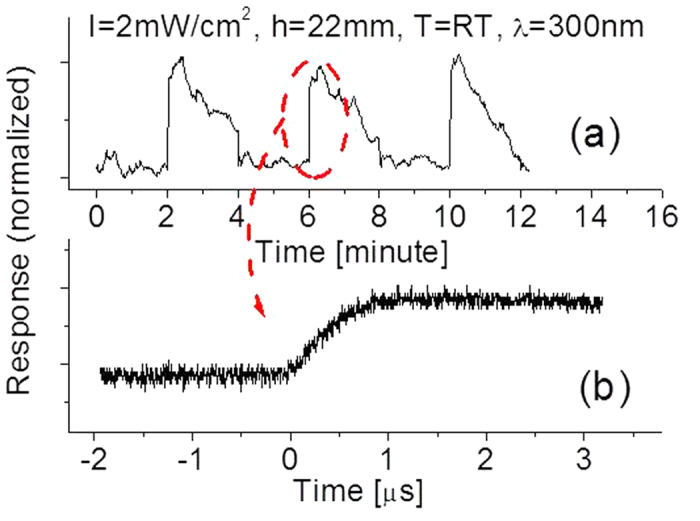
Relationships between original signal, thermal effect and pure signal/responses from the fabricated detector exposure to 300 nm UV light with intensity of 2 mW/cm^2^.

The mechanism responsible for the response-recovery times of the detector is difficult to determine because it depends on many factors such as the nature of samples, light wavelength, doping, doping element and concentration, operating temperature, electrode arrangement, and humidity content, among others. We have not yet fully understood the basic mechanism that causes large differences of time evolutions of response curves for the detector exposed to 250, 300, and 350nm UV light sources, respectively. Here it should be mentioned that the effect of electromagnetic wave noise caused by turning on or off the switcher of electrical power was not the cause for the phenomena described above. The evidence is that a similar phenomenon was still visible, as shown in Fig. 11a, where the prototype was cycled between the “switch-on” and “switch-off” of the 300 nm UV light sources by using a shutter so that possible electromagnetic wave effect from the switcher on the response signal during cyclic test has completely been removed. Clearly, the quick change of the response at the edge of very beginning and at the end of each cycle could be due to the quick change of the incident light intensity.

The response time of photodetector has been measured by using LeCroy 500 MHz oscilloscope. A typical result is shown in Fig. 11b, from which it is easily estimated that the response time of the detector is only 1 μs. This is a quite short response time for the MSM architecture based photodetector. Actual response time and recovery time might be shorter. This is because time delay for reaching full intensity for UV lamps after switching on the lamp, and florescence after switching off the lamp would affect the measurement results.

## Conclusions

The obtained experimental data clearly indicated that our approach described above can be used for quick fabrication of high-performance deep UV photodetectors. The fabricated detectors appear to have a very stable baseline and repeatability to wide regions of deep UV lights with wavelengths from 250 nm to 350 nm. At 2 mW/cm^2^ 250 nm light radiation, the yielded photocurrent is 55 μA. Correspondingly, a detector responsivity of up to 0.18 A/W was obtained. This value is almost 40% higher compared to commercial detectors. After increasing the operating temperature to 300 °C, the fabricated detector still runs well with good features in stability and repeatability. This characteristic is much better than that of most commercial SiC detectors that normally operate at temperatures less than 150 °C. However, once the operating temperature reaches 400 °C, the signal-to-noise ratio is seriously decreased and the thermal noise nearly dominates the output. At this moment, it is difficult to identify the real photocurrent signal from the detector output.

It was also found that the output signal strength is slightly affected by bias and that humidity has the effect of increasing the response and recovery times of the detector. Therefore, when the detector is exposed to deep UV light source, the vaporization of partially adsorbed water molecules from the surface resulted in the recovery of the sensitivity and faster response and recovery times.

From the obtained experimental data, we can conclude that the response strength of the detector exposed to 300–350 nm lights is almost 7–8 times less than that to 250 nm light at the same radiation intensity. Furthermore, the evolutions of the responses during the cyclical test by using 300 and 350 nm lights were also obviously different from that of the detector to 250 nm light. The fastest response time observed was only 1 μs under 300 and 350 nm wavelengths of incident radiation.

## Additional Information

**How to cite this article**: Aldalbahi, A. *et al*. A new approach for fabrications of SiC based photodetectors. *Sci. Rep.* **6**, 23457; doi: 10.1038/srep23457 (2016).
